# ART achievements and challenges in Africa

**DOI:** 10.1186/1758-2652-13-S4-K3

**Published:** 2010-11-08

**Authors:** J Hakim

**Affiliations:** 1 University of Zimbabwe, Dept of Medicine, Harare, Zimbabwe

## 

Combination antiretroviral therapy (ART) as we know it today has only been practiced in Africa in a significant segment of people living with HIV (PLH) in the past 6 years. We have put behind us the litany of reasons which were given as obstacles in the implementation of ART in Africa. Today the majority of the 5.2 million people on ART are from sub-Saharan Africa. Cohort comparisons between resource-rich and resource-limited settings have demonstrated similar immunologic and virologic responses to ART. There is however a higher mortality, of more than 4 times in the first 6 months of ART in the developing world. There is therefore need to address this early mortality to fully harness the benefits of ART. Ecological data from a region in South Africa has shown that tuberculosis rates have fallen by 50% in the community in association with the widespread use of ART. The benefits of ART extend to many other spheres including reduction of perinatal infection and improved socio-economic indices in children and adults. There is also strong argument that the increased roll-out of ART will impact maternal mortality, hence contribute to the achievement of MDG5. Potential additional benefits of antiretroviral therapy in prevention are emerging as exemplified by the positive outcome of CAPRISA004; a study that showed a preventive role of tenofovir gel of 39% in women in Durban, South Africa. Other ARV-based microbicide studies and studies of ART as pre-exposure prophylaxis will report soon and may broaden the use and benefits of ART beyond therapeutics. The roll-out of ART in Africa is not without challenges. Currently only 42% of PLH who qualify for treatment are on therapy, moreover it is estimated that 10 million more will require ART when the new WHO guidelines recommendation of ART initiation at a threshold of 350 cells/mm^3^ are considered.

Other recommendations including emphasis on diagnosing HIV early, the use of more expensive drugs in first-line therapy, the emphasis on immunologic and virologic diagnosis of failure will require increased resources. The availability of resources is threatened by the world economic crisis and changing funding paradigms. The challenge ahead is to mobilize resources to enable use of ART in an efficient manner and in a way that exploits the expanding role of ART in both HIV therapy and prevention.

